# Developing an AI algorithm to detect predictors of poor performance in a self‐administered, web‐based digital biomarker for Alzheimer’s Disease: proof of concept

**DOI:** 10.1002/alz.092887

**Published:** 2025-01-09

**Authors:** Joe Butler, Adewale Samuel Owo, Tamlyn J Watermeyer, Samuel O. Danso, Mario A Parra‐Rodriguez, Binding in Neuropsychiatric Disorders (BIND) Group

**Affiliations:** ^1^ University of Sunderland, Sunderland United Kingdom; ^2^ Female Brain Health and Endocrine Research (FemBER) Consortium, Newcastle, Edinburgh, London United Kingdom; ^3^ Faculty of Health & Life Sciences, Northumbria University, Newcastle United Kingdom; ^4^ Edinburgh Dementia Prevention, Centre for Clinical Brain Sciences, College of Medicine and Veterinary Medicine, University of Edinburgh, Edinburgh United Kingdom; ^5^ University of Edinburgh, Edinburgh United Kingdom; ^6^ University of Strathclyde, Glasgow United Kingdom

## Abstract

**Background:**

The Visual Short Term Memory Binding (VSTMBT) task is a gold‐standard cognitive assessment for the identification of Alzheimer's Disease and associated risk factors, including during the preclinical stage. Previous work from our group (Butler, Watermeyer, …& Parra 2024) demonstrated in a small number (n=37) of healthy older adults that data collected using a web‐based, self‐administrated version of the task provides data comparable to that collected in laboratory conditions. Here we incorporated a machine learning (ML) approach to explore impacts of risk factors on this task in a larger digital dataset.

**Method:**

Using data (n=359) collected from an online study incorporating the VSTMBT and lifestyle, psychological, and health data, we created a Binding Cost score which has shown to approximate AD‐related neuropathology (Parra et al., 2024). This categorised participants as either strong‐binders (SB – indicative of no pathology; 85.9% percent of the sample) or weak‐binders (WB – indicative of pathology; 14.1%).

We trained three ML algorithms (Random Forest (RF), K‐Nearest Neighbour (KNN) and Decision Tree (DT) by employing SMOTE technique to overcome the imbalance in group distribution. We applied a 10‐fold cross‐validation with hyper‐parameter tuning to optimise the models based on the selected variables (including age, sex, education, BMI, loneliness, and existing‐morbidities) to predict individual’s risk of cognitive impairment based on the groupings (SB vs WB). Models’ performances were examined on 20% of unseen test set.

**Result:**

Aside from existing morbidities, which were higher in weak binders (WB = 0.41 (sd+2=0.79); SB =0.22(sd+2=0.49); t=2.21; p=0.03), other measures did not differ between groups. Regarding performance of the ML models, RF achieved the best performance (accuracy: 91%; recall=91%; precision=91%; AUC=97%) compared to KNN (accuracy: 81%; recall=81%; precision=84%; AUC=91%) and DT (accuracy: 81%; recall=81%; precision=82%; AUC= 85%). Feature importance analysis of the RF model suggests mental health, BMI, and fatigue have the highest impact on the prediction model, while sex and multi‐morbidity score have the least impact.

**Conclusion:**

The study underscores the potential of web‐based cognitive assessments and ML for remote monitoring and early identification of AD risk factors, contributing to the advancement of accessible tools for early detection.

